# Peptides Derived from *Rhopilema esculentum* Hydrolysate Exhibit Angiotensin Converting Enzyme (ACE) Inhibitory and Antioxidant Abilities

**DOI:** 10.3390/molecules190913587

**Published:** 2014-09-02

**Authors:** Jun Li, Qian Li, Jingyun Li, Bei Zhou

**Affiliations:** State Key Laboratory of Reproductive Medicine, Department of Plastic & Consmetic Surgery, Nanjing Maternity and Child Health Hospital, Nanjing Medical University, Nanjing 210029, China; E-Mails: drliqian@126.com (Q.L.); lijingyun175@gmail.com (J.L.); enzhoubei@126.com (B.Z.)

**Keywords:** jellyfish (*Rhopilema esculentum*), ACE inhibition, antioxidant, endothelial cells, molecular docking

## Abstract

Jellyfish (*Rhopilema esculentum*) was hydrolyzed using alcalase, and two peptides with angiotensin-I-converting enzyme (ACE) inhibitory and antioxidant activities were purified by ultrafiltration and consecutive chromatographic methods. The amino acid sequences of the two peptides were identified as VKP (342 Da) and VKCFR (651 Da) by electrospray ionization tandem mass spectrometry. The IC_50_ values of ACE inhibitory activities of the two peptides were 1.3 μM and 34.5 μM, respectively. Molecular docking results suggested that VKP and VKCFR bind to ACE through coordinating with the active site Zn(II) atom. Free radical scavenging activity and protection against hydrogen peroxide (H_2_O_2_)-induced rat cerebral microvascular endothelial cell (RCMEC) injury were used to evaluate the antioxidant activities of the two peptides. As the results clearly showed that the peptides increased the superoxide dismutase (SOD), catalase (CAT) and glutathione peroxidase (GSH-px) activities in RCMEC cells), it is proposed that the *R. esculentum* peptides exert significant antioxidant effects.

## 1. Introduction

The jellyfish *Rhopilema esculentum* is a commercially available species along the coast of China and has long been used as both food and medicine. *R. esculentum* has been recognized for its beneficial properties since the time of the Qing dynasty. It was recorded that *Xuegeng* decoction contained *R. esculentum* and *Eleocharis dulcis* and was used as a treatment for essential or primary hypertension. Recent studies have shown that *R. esculentum* contains proteins and peptides [1], which possess various bioactivities, including antioxidant [2], anti-hypertension [3], and neurotoxicity effects [4]. Protein hydrolysate from *R. esculentum* also exhibits angiotensin-I-converting enzyme (ACE) inhibitory activity and significantly reduces the blood pressure of spontaneously hypertensive rats [3]. In addition, *R. esculentum* hydrolysate also has strong free radical scavenging ability, and therefore, antioxidant activity [1].

In recent decades, marine life or food-derived protein hydrolysate peptides have attracted significant attention owing to their diverse biological functions and safety. It has reported that these peptides possess ACE inhibitory [5], antioxidant [6], immunomodulatory [7], and antimicrobial activities [8]. Studies showed that some peptides from food sources, such as flaxseed protein [9], protein isolate from pumpkin oil cake [10] exert both ACE inhibitory and antioxidant activities. Several natural ACE inhibitory and antioxidant peptides have been purified from marine organisms, such as *Okamejei* [11], shrimp [12], and clam [13].

In the process of hypertension, ACE plays an important role in regulating blood pressure, and ACE inhibitors are considered to be one of the therapeutic methods for treating anti-hypertension. Several ACE inhibitors, including captopril, lisinopril, and enalapril, are synthetic molecules which are clinically used as anti-hypertension agents. Oxidation is an essential reaction in all living organisms. The formation of reactive oxygen species (ROS) and free radicals is unavoidable during the oxidative metabolic process. Overproduction of ROS is believed to be involved in many diseases, including hyperthermia, hypertension, and neurodegenerative disorders [14,15,16]. In organisms, antioxidant enzymes, such as superoxide dismutase (SOD), catalase (CAT) and glutathione peroxidase (GSH-px), protect cells or tissues from injury. Some synthetic antioxidant agents, such as butylated hydroxyanisole (BHA) and butylated hydroxytoluene (BHT) are commonly used as free radicals in food and biological systems.

The anti-hypertensive drugs or antioxidants are often associated with undesirable side-effects, such as angioedema [17]. Thus investigators have increased preference for natural food-derived ACE inhibitors or antioxidants [18]. Protein hydrolysate or peptides with both ACE inhibitory and antioxidant activities might be helpful in treating hypertension. And due to their minimal side-effects and various bioactivities, these natural protein hydrolysate or peptides have recently been the focus of considerable research interest. In the present study, fractions and purified peptides from *R. esculentum* displaying both ACE inhibitory and antioxidant activities were obtained. The main objectives of the present investigation were investigating the protective effects of these peptides against oxidative stress injury on microvascular endothelial cells. Moreover, molecular docking was applied to reveal the mode of interaction between peptides and ACE.

## 2. Results and Discussion

### 2.1. Isolation, Purification and Characterization of Peptides

**Figure 1:**
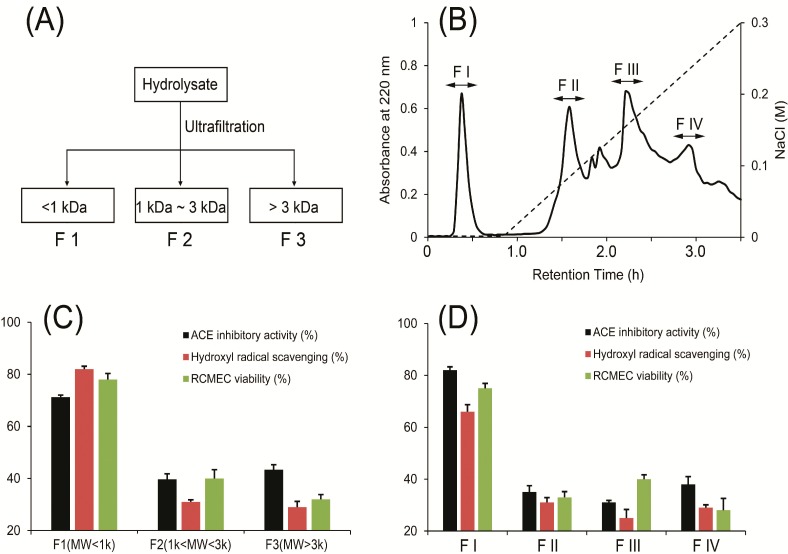
(**A**) Separation of peptides from *R. esculentum* hydrolysate via ultrafiltration. (**B**) Anion exchange chromatogram of F1 obtained by ultrafiltration on a DEAE Sepharose Fast Flow. (**C**) ACE inhibitory and antioxidant activities (hydroxyl radical scavenging activity and protection of rat cerebral microvascular endothelial cell (RCMEC) against H_2_O_2_) of F1, F2 and F3. (**D**) ACE inhibitory and antioxidant activities (hydroxyl radical scavenging activity and protection of RCMEC against H_2_O_2_) of F I, F II, F III and FIV.

**Figure 2:**
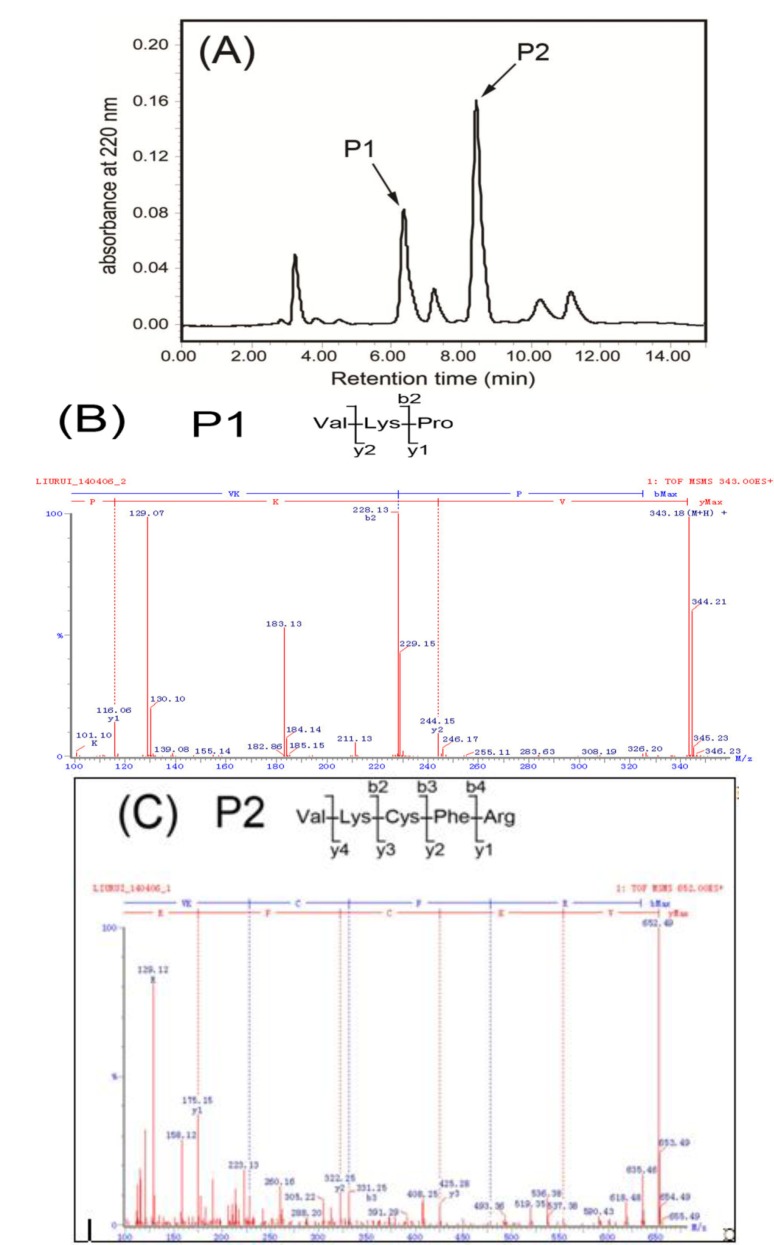
(**A**) Reversed-phase HPLC separation pattern on a C_18_ column of the active fraction FI from Figure 1D. The gradient conditions were: 0–1.5 min, 2% methanol; 1.5–10 min, linear from 2% to 30% methanol. Elutions were performed at a flow rate of 0.5 mL/min using a UV detector at 220 nm. (**B**) Identification of molecular mass and amino acid sequences of P1, MS/MS experiments were performed on ESI Q-TOF MS. (**C**) Identification of molecular mass and amino acid sequences of P2, MS/MS experiments were performed on ESI Q-TOF MS. P1 and P2 were sequenced via *de novo* sequencing using Biolynx software.

*R. esculentum* alcalase hydrolysate was separated into three fractions (F1–F3) using molecular weight-based ultrafiltration (Figure 1). The fraction composition of the hydrolysate was ~31% F1 (MW < 1 kDa), 24% F2 (1 kDa < MW < 3 kDa), and 45% F3 (MW > 3 kDa). Accordingly, the fraction with the strongest activity was further separated via DEAE Sepharose Fast Flow ion-exchange column chromatography into four fractions (FI–FIV). Two peptides, P1 and P2, were further purified from FI using a Waters AutoPurification high performance liquid chromatography (HPLC) system, as shown in Figure 2. Using ESI Q-TOF MS, the amino acid sequences of P1 and P2 were identified as VKP (342 Da, P1) and VKCFR (651 Da, P2), respectively.

Molecular weight, amino acid constituents and sequences are important factors that determine peptide bioactivity. Peptides with molecular weights lower than 1 kDa usually possess higher ACE inhibitory activity than other fractions [19]. Lower molecular weight peptides have additionally been reported to exert strong antioxidant effects. In the present study, active peptides were enriched initially via ultrafiltration. From F1 showing the highest ACE inhibitory and antioxidant activities among the three fractions, two peptides displaying both activities were purified using ion-exchange and reversed-phase chromatography.

The amino acid sequences of the peptides were analyzed via LC-MS/MS. As shown in Figure 2B, a tripeptide with the sequence VKP was identified based on the *m/z* 343.18 ion, which showed a y ion series of *m/z* 116.06 and *m/z* 244.15, and a b ion of *m/z* 228.13. Similarly, a pentapeptide with a primary sequence “VKCFR” was purified and identified (Figure 2C). The VKCFR sequence was calculated based on a y ion series of *m/z* 175.15, *m/z* 322.25, *m/z* 425.28, *m/z* 553.38 and a b ion series of *m/z* 228.21, *m/z* 331.25, and *m/z* 478.32 in the MS/MS spectrum.

### 2.2. Antioxidant and ACE Inhibitory Activities of Purified Peptides

In Section 2.1, F1–F3 were firstly isolated and the ACE inhibitory acitivities of F1–F3 (100 μg/mL) were measured at 71.2% ± 0.8%, 39.7% ± 2.1%, and 43.4% ± 1.9%, respectively. Thus, among the three fractions, F1 showed the strongest ACE inhibitory activity. Moreover, F1 exhibited the strongest antioxidant activity, both in the hydroxyl radical scavenging and RCMEC 3-(4,5-dimethylthiazol-2-yl)-2,5-diphenyltetrazolium bromide (MTT) assays. Secondly, among the separated fractions from anion exchange chromatogram, FI showed significant high ACE inhibitory and antioxidant activities.

**Figure 3:**
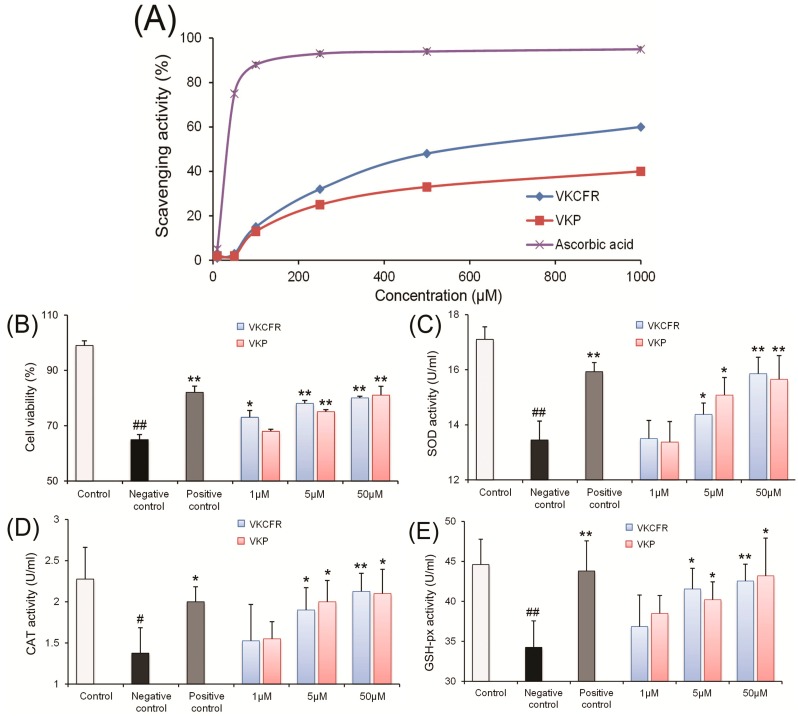
(**A**) Hydroxyl radical scavenging activity of VKP and VKCFR. (**B**) Protective effects of VKP and VKCFR pretreatment at different concentrations (1, 5, and 50 μM) on RCMEC exposed to 300 μM H_2_O_2_ in the MTT assay. (**C**) Effects of VKP and VKCFR treatment at different concentrations (1, 5, and 50 μM) on SOD enzymatic activity in RCMEC exposed to H_2_O_2_. (**D**) Effects of VKP and VKCFR treatment at different concentrations (1, 5 and 50 μM) with H_2_O_2_ exposure on RCMEC CAT enzymatic activity. (**E**) Effects of H_2_O_2_ exposure with VKP and VKCFR treatment at different concentrations (1, 5, and 50 μM) on RCMEC GSH-px enzymatic activity. Significant difference compared to the control at # *p* < 0.05, ## *p* < 0.01; significant differences compared to the negative control at * *p* < 0.05, ** *p* < 0.01, *n* = 6 in each group.

Antioxidant activity tests were performed with the two peptides. The hydroxyl radical scavenging assay was used to evaluate the ability of peptides to scavenge free radicals. As seen in Figure 3A, both purified peptides possessed the ability to quench the hydroxyl radical. The MTT assay was additionally used to assess the effects of P1 and P2 on H_2_O_2_-induced RCMEC injury. After treatment with 5 and 50 μM P1 and P2, cell viability was significantly increased (Figure 3B). Upon preincubation with 1, 5 and 50 μM P1 and P2, the activities of the antioxidant enzymes, SOD, CAT and GSH-px, in RCMEC significantly increased in a dose-dependent manner as depicted in Figure 3C–E, respectively.

**Figure 4:**
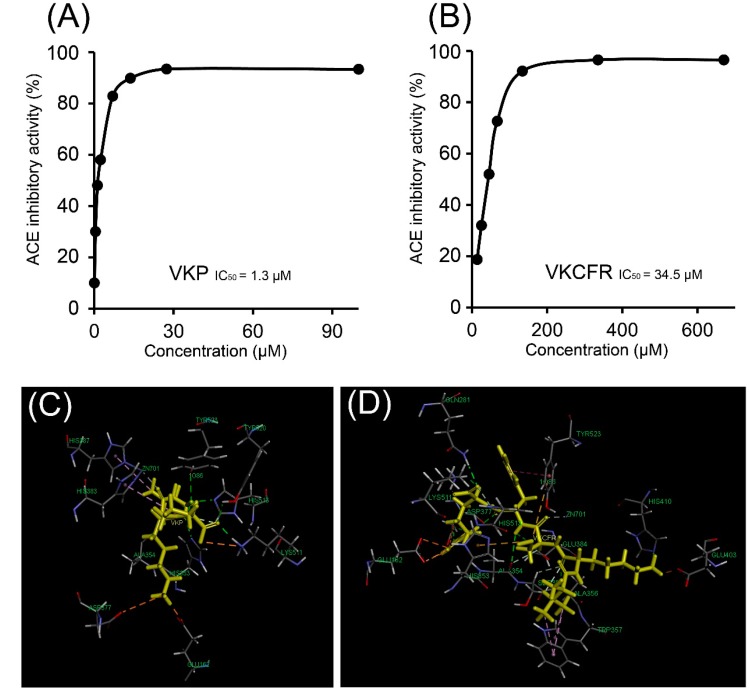
(**A**) ACE inhibitory activity of VKP with IC_50_ of 1.3 μM. (**B**) ACE inhibitory activity of VKCFR with IC_50_ of 34.5 μM. (**C**) Interface overview of optimal docking positions of VKP at the ACE catalytic site. (**D**) Interface overview of optimal docking positions of VKCFR at the ACE catalytic site.

The two peptides were chemically synthesized and their ACE inhibitory and antioxidant activities were examined. As shown in Figure 4A,B, the VKP tripeptide displayed the most potent ACE inhibitory activity with an IC_50_ value of 1.3 μM, while the IC_50_ value of VKCFR activity was determined as 34.5 μM.

ACE inhibitory and antioxidant activities were related to the amino acid sequences of the peptides [20]. Residues, such as Val, Pro, Cys, and Phe normally exist in ACE inhibitory peptides [21]. A tripeptide with Pro at the carboxy-terminus has been identified as a potent ACE inhibitor [22]. The thiol group of Cys might play an important role in binding of peptides to the ACE active site [23]. Moreover, ACE binding is strongly influenced by the C-terminal sequence of the peptide, and hydrophobic residues in this region, such as Leu, Pro, Phe, Trp, and Tyr, significantly increase ACE binding affinity [24]. Additionally, high content of the amino acids Val, Leu, and Tyr at the N-terminus enhances the ACE inhibitory activity of peptide [25,26]. In the present study, the two active peptides contained Val residues at the N-terminus and Pro, Phe or Arg at the C-terminus, which might contribute to the ACE inhibitory activity.

The presence of hydrophobic residues in peptides could enhance their biological activities, such as antioxidant and ACE inhibitory properties [19]. In particular, the ability of hydrophobic amino acids to increase binding affinity or solubility in lipid may facilitate peptide approach to ACE or the cell membrane [20]. Hydrophobic amino acids in VKP and VKCFR, such as Pro, Phe, Val and Cys, may enhance both ACE inhibitory and antioxidant activities. Cys, Phe and Arg residues in the two peptides may act as proton donors to scavenge radicals and protect cells against H_2_O_2_-induced injury.

In the present study, a model of H_2_O_2_-induced endothelial cell injury was used to evaluate the antioxidant activity of peptides. Recent evidence indicates that the oxidative stress leads to excessive ROS in hypertensive patients and animal models [27]. Oxidative stress also plays a key role in the pathogenesis of hypertension and ROS acts as a vasoconstrictive mediator induced by angiotensin II, endothelin-1 and urotensin-II. Vasculature has been established as a rich source of ROS generated in endothelial cells [28]. Thus, antioxidant activity and protection against H_2_O_2_-induced injury in endothelial cells may indirectly relieve angiotonia. The enzymatic antioxidants, SOD, GSH-px and CAT, play an important role in protecting against oxidative stress injury. SOD catalyzes the degradation of O_2_^−^ to O_2_ and H_2_O_2_, CAT converts H_2_O_2_ into H_2_O and O_2_, and GSH-px reduces both H_2_O_2_ and organic hydroperoxides. In the current study, both purified peptides displayed antioxidant activity. The two peptides not only quenched the hydroxyl radical directly due to proton donor ability, but also increased antioxidant enzyme activity, ultimately contributing to protect endothelial cells against oxidative damage.

### 2.3. Molecular Docking of ACE and Peptides

Studies on the docking of VKP and VKCFR at the ACE catalytic site in the presence of the cofactor Zn(II) disclosed the best position (Figure 4). ACE is a metalloenzyme with a zinc ion in the active site that coordinates with His383, His387, and Glu411. The docking results demonstrated that VKP and VKCFR effectively bind ACE. The scores presented in Table 1 show the different binding energies of VKP and VKCFR. As shown in Figure 4C,D, VKP may form hydrophobic interactions with His383 and His387 as well as hydrogen bonds with His513, His353, Lys511, Tyr520, Tyr523, and Ala354. VKCFR may form hydrophobic interactions with Tyr523 and Trp357 and hydrogen bonds with residues Gln281, Lys511, His513, Asp377, Glu384, Ala354, Ser355, Ala356, and His410. The thiol group of cysteine in VKCFR can also form sulfur bonds with Tyr523 and His353. Molecular docking can be effectively applied to study the structure-activity relationships between bioactive peptides and ACE [29,30]. Data from the molecular docking studies collectively indicate that the two active peptides bind the catalytic pocket of ACE through a network of hydrogen bonds and hydrophobic interactions. Van der Waals, electrostatic, and potential energy values are listed in Table 1. Interactions between ACE inhibitory peptides and Zn (II) at the active site play a significant role in ACE deactivation. Both VKP and VKCFR appear to be positioned to coordinate with the active site Zn(II) atom.

**Table 1:** Energy results of molecular docking (kJ/mol).

Ligand	Van der Waals Energy	Electrostatic Energy	Potential Energy	Bond Energy	CHARMm Energy
VKP	−8.347	−52.149	−26.028	1.296	−26.028
VKCFR	−17.823	−134.961	−132.409	1.988	−132.409

## 3. Experimental Section

### 3.1. Materials

*R. esculentum* jellyfish was collected from marine aquaculture farms of Nanjing, Jiangsu Province, China, in July 2013, and immediately stored at −20 °C. Alcalase (10^4^ U/g), angiotensin I-converting enzyme (ACE), hippuryl-histidyl-leucine (HHL), and hippuric acid (HA) were purchased from Sigma Chemical Co. (St. Louis, MO, USA). HPLC-grade acetonitrile (ACN) and trifluoroacetic acid (TFA) were obtained from Tedia Company Inc. (Fairfield, CA, USA). All other reagents were acquired from Sigma Chemical Co. All reagents were of analytical grade.

### 3.2. Preparation of Hydrolysate

One kilogram wet sample of *R. esculentum* with moisture and protein contents of about 91% (w/w) and 5% (w/w), respectively. The sample was washed with water three times to remove salt, cut into small pieces and boiled twice with water (1/10, w/v) for 30 min each. After boiling, the sample was cooled down to room temperature (25 °C), then sieved to separate the undissolved mass from the soluble fraction. The insoluble residue was then hydrolyzed by incubating with alcalase in 0.1 M sodium phosphate buffer (PBS, pH 8.0 at sample/solvent ratio of 1/10) for 2 h at 45 °C at an enzyme-to-substrate ratio of 2% (w/w). After incubation, the mixture was inactivated by incubating at 85 °C for 10 min. Based on the method of Qian [31], the degree of hydrolysis was determined by measuring the nitrogen content soluble in 10% trichloroacetic acid, and the degree of hydrolysis was estimated to be 63.5%. Hydrolysate was lyophilized and stored at −20 °C for less than 2 months.

### 3.3. Assay for ACE Inhibitory Activity

ACE inhibitory activity was measured as follow: sample solution was firstly centrifuged at 10,000 rpm, and then 10 μL sample solution (with various concentrations, 50 mM borate, pH 8.3) was added to 30 μL 2.5 mM HHL solution and incubated for 5 min at 37 °C. Subsequently, 20 μL of 0.1 U/mL ACE solution (50 mM borate, 0.3 M NaCl, pH 8.3) was added, and the mixture was incubated at 37 °C for 60 min. The reaction was stopped by adding 70 μL 1 M HCl. A blank sample of buffer (50 mM borate, pH 8.3) alone was used as the negative control. Samples were filtered through a 0.45 μm nylon syringe filter and separated on a C18 column (4.6 mm × 150 mm, 5 μm). HA and HHL were detected via the measurement of absorbance at 228 nm. The column was eluted at a flow rate of 0.8 mL/min using a mobile phase composed of (A) 0.05% (v/v) TFA in water and (B) 0.05% (v/v) TFA in acetonitrile. Elution was carried out using a gradient of 10%–60% B in the first 10 min, followed by 60%–10% B in the next 2 min. HA was quantified by integration of peak areas (A). Percentage ACE inhibition was calculated according to the following equation:

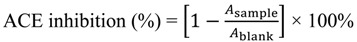
(1)
where A_Sample_ and A_blank_ are peak areas corresponding to HA for an inhibitor sample and the blank sample, respectively.

### 3.4. Determination of Antioxidant Activities

#### 3.4.1. Hydroxyl Radical Scavenging Assay

In a test tube, sodium phosphate buffer (0.4 mL, 50 mM, pH 7.5), sample solution (0.1 mL) of various concentrations, 1 mM ethylenediaminetetraacetic acid (EDTA, 0.1 mL), 10 mM H_2_O_2_, 60 mM 2-deoxy-d-ribose (0.1 mL of peptide solution in place of 2-deoxy-d-ribose solution as a sample blank), 2 mM ascorbic acid, and 1 mM FeCl_3_ were added, then incubated at 37 °C for 1 h. The reaction was terminated by adding 8 M HCl (1 mL). Subsequently, 1 mL of 1% (v/v) thiobarbituric acid (TBA) was added to the reaction tubes, which were placed in boiling water for 15 min, and the absorbance was read at 532 nm. The free radical scavenging effects were calculated according to the following formula: hydroxyl radical scavenger = (A_0_ − A)/A_0_ × 100%, where A_0_ and A represent the absorbance of the control and sample, respectively.

#### 3.4.2. Cell Culture and Cytotoxicity Determination

The H_2_O_2_-induced rat cerebral microvascular endothelial cells (RCMEC) injury model was used to evaluate the antioxidant activity of samples [32]. The isolation and the culture of RCMEC was performed according to the method of Liu [2], with slight modifications. Sprague-Dawley neonatal rats (7–10 days old) were used for RCMEC isolation. Briefly, isolated cerebral gray matter was digested with trypsin (0.05%) at 37 °C for 20 min, filtered through a 149 µm nylon mesh, and the filtrate was collected. The filtrate was re-filtered through a 79 µm nylon mesh, and the retentate was collected and digested with collagenase type II (0.1%) at 37 °C for 25 min. Then the digested retentate was centrifuged at room temperature for 5 min (200× *g*), and the precipitate with cells were collected. Cells were cultured in Dulbecco’s Modified Eagle Medium (DMEM) and F-12 supplemented with 20% newborn calf serum at 37 °C with 5% CO_2_, with daily medium replacement, until confluency (11–14 days). About 10 mL cells suspension was plated at the appropriate density to the cell culture bottle according to each experimental scale.

Animal welfare and experimental procedures were strictly in accordance with the Guide for the Care and Use of Laboratory Animals (US National Research Council, 1996) and the related ethics regulations of the hospital in which this study was conducted.

Cytotoxic effects of samples on cells were measured by MTT assay, as follows: RCMEC cells were seeded in 96-multiwell plates to a final concentration of 2 × 10^4^ cells per well and maintained until sub-confluence. Cells were incubated with hydrolysate fractions or purified peptides at different concentrations for 12 h, prior to the addition of 300 µM H_2_O_2_. After removing medium and washing with PBS (pH 7.8) twice, fresh low serum (5%) medium containing 300 µM H_2_O_2_ was added to cells, followed by incubation at 37 °C, 5% CO_2_ for 2 h. At the indicated times, 100 µL serum-free culture medium and 20 µL MTT PBS solution (5 mg/mL) was added in each well. After 4 h of 37 °C incubation, 120 µL cell lysis solution (10%, w/v SDS, 5%, v/v isobutanol, 12 mM HCl) was added to each well and the plate was incubated at 37 °C overnight. The plates were measured directly in the plate reader at 570 nm and a reference wavelength of 630 nm. The MTT assay is used as an index of cell survival [33]. MTT is an indicator of mitochondrial activity of living cells. MTT is reduced to a colored compound (formazan) by mitochondria.

#### 3.4.3. Determination of Antioxidant Enzyme Activity

Superoxide dismutase activity was estimated using the following method. Cell culture supernatant (~200 μL) was mixed with sodium pyrophosphate buffer, phenazine methosulphate (PMT) and nitro blue tetrazolium (NBT). The reaction was initiated by adding NADH and incubating at 37 °C for 40 min and stopped by glacial acetic acid, followed by measurement at 560 nm. Units of SOD activity were expressed as the amount of enzyme required to inhibit reductase of NBT by 50%.

The assay mixture for catalase activity measurement consisted of PBS (1.95 mL, 50 mM, pH 7.0), H_2_O_2_ (1.0 mL, 0.019 M), hepatic PMS (0.05 mL, 10%, w/v) in a final volume of 3.0 mL. Changes in absorbance were recorded at 240 nm. CAT activity was calculated in terms of nmol H_2_O_2_ consumed per minute.

GSH-px was measured in a reaction using dithiobisnitrobenzoic acid (DTNB), a symmetric aryl disulfide. DTNB reacts with the free thiol to generate a mixed disulfide plus 2-nitro-5-thiobenzoic acid (TNB), quantified by absorbance at 412 nm. GSH-px activity was determined by quantifying the catalyzed reaction rate of H_2_O_2_ and GSH. One unit (U) of GSH-px was defined as the amount that reduced the GSH level for 1 μM calculated as U/mL or U/mg protein.

### 3.5. Purification of Bioactive Peptides

#### 3.5.1. Ultrafiltration

Ultrafiltration was performed using membranes with 1 and 3 kDa cut-off values on a Mini Pellicon Ultrafiltration system (Millipore Corporation, Billerica, MA, USA). *R. esculentum* hydrolysate prepared as described above were passed through a 0.45 μm nylon syringe filter and separated into three fractions with increasing molecular weights via ultrafiltration: Fraction 1 (F1, MW < 1 kDa), Fraction 2 (F2, 1 kDa < MW < 3 kDa), and Fraction 3 (F3, 3 kDa < MW). F1, F2 and F3 were lyophilized and subjected to the ACE inhibitory activity test and antioxidant determination, as described above.

#### 3.5.2. Ion-Exchange Chromatography

The active fraction after ultrafiltration was dissolved in 50 mM PBS (pH 7.8) and loaded onto a 2-Diethylaminoethanol (DEAE) Sepharose Fast Flow ion-exchange column (2.0 × 20 cm; GE Healthcare, Menlo Park, CA, USA) previously equilibrated with 50 mM PBS (pH 7.8), followed by elution with a linear gradient of 0–0.3 M NaCl at a 1 mL/min flow rate. The eluted solution was monitored at 214 nm and the main peak was collected. Fractions I, II, III and IV were lyophilized and subjected to the ACE inhibitory activity test and antioxidant determination, as described above.

#### 3.5.3. RP-HPLC

Active fractions exhibiting ACE inhibitory and antioxidant activities after ion-exchange chromatography were further separated with the RP-HPLC system (Waters 2545-2489-2676 AutoPurification HPLC system, Waters Corporation, Milford, MA, USA, SunFire C_18_ Column, 19 mm × 50 mm, 5 μm). Eluting solvent A: 0.1% (v/v) TFA in pure water and B: 0.1% (v/v) TFA in methanol. Elution was carried out with 2% B in 1.5 min and a linear gradient from 2% to 60% B in another 10 min at a flow rate of 15 mL/min. The main peak, representing pure peptides, was collected. The purity and molecular weight of peptides were determined using ultra performance liquid chromatography electrospray ionization tandem mass spectrometry (UPLC ESI-MS/MS).

### 3.6. Characterization of Purified Peptides

A Waters ACQUITY UPLC system, coupled with a Synapt Mass Quadrupole Time-of-Flight Mass Spectrometer (UPLC-ESI Q-TOF MS), was used to identify the purified peptides. Purified peptides were dissolved in mobile phase, and 5 μL of peptide solutions loaded onto an ACQUITY BEH C_18_ column (100 mm × 2.1 mm, 1.7 μm). Elution was performed using a mobile phase consisting of water-acetic acid (100:0.5, v/v) (eluent A) and methanol (eluent B). Gradient elution was performed as follows: 0–3 min, 5% B; 3–15 min, 5%–30% B; 15–17 min, 30% B; flow rate: 0.3 mL/min. MS analysis was operated in the positive electrospray ionization mode. MS/MS analyses were performed using CID. Collision energy was selected from 10 to 35 eV. Argon was introduced as the collision gas at a pressure of 10 psi. Sequencing of peptide was acquired over the *m/z* range 50–2000 using the Biolynx software.

### 3.7. Peptide Synthesis

ACE inhibitory peptides screened via molecular docking were synthesized using the solid-phase method and purified via HPLC after deprotection. These experiments were performed by the GenScript Corporation (Nanjing, China).

### 3.8. Molecular Docking Analysis

The crystal structure of human ACE complexed with lisinopril was retrieved from the Brookhaven Protein Data Bank with the structure labeled “1O86” [34]. This crystal structure was used as the “receptor” in present molecular docking studies. The structures of peptides identified via LC-MS/MS from active hydrolysate were generated with Accelrys Discovery Studio 4.0 software, and the energy minimized with the CHARMm program using steepest descent and conjugate gradient techniques. The Binding Site tool was applied for active site determination between ACE and peptides. Docking runs were performed with a radius of 4 Å, and molecular docking evaluation performed according to the scores of several functions. The optimal conformations were used to calculate the binding energy value.

### 3.9. Statistical Analysis

Data are presented as mean values ± S.D., and analyzed using Student’s *t*-test. Differences with *p*-values < 0.05 were considered statistically significant. ACE inhibitory activity assay was determined for 4 replicates in each concentrations, IC_50_ values were calculated using the GraphPad software. Experiment of radical scavenging assay was performed for four replicates at each concentration. MTT assay and determination of antioxidant enzyme activity were performed for six replicates.

## 4. Conclusions

ACE inhibitory peptides derived from natural sources have significant therapeutic potential as anti-hypertensive agents with few adverse side-effects. *R. esculentum* hydrolysate were used as a biological source of ACE inhibitory peptides. Along the coast of China, *R. esculentum* were commercially available species and proved to be a rich source of peptides. In the present study, the identified peptides, VKP and VKCFR, were synthesized, and their ACE inhibitory and antioxidant activities further determined. Both peptides showed ACE inhibitory activity with IC_50_ values of 1.3 μM and 34.5 μM, respectively. Results from docking simulation studies suggested that the peptides bind ACE through coordination with the active site Zn(II) atom. The H_2_O_2_-induced endothelial cell injury model was used to evaluate the antioxidant activity of peptides. The peptides clearly protected endothelial cells against oxidative damage, possibly via quenching the hydroxyl radical and enhancing antioxidant enzyme activity.

## Figures and Tables

**Figure A1:**
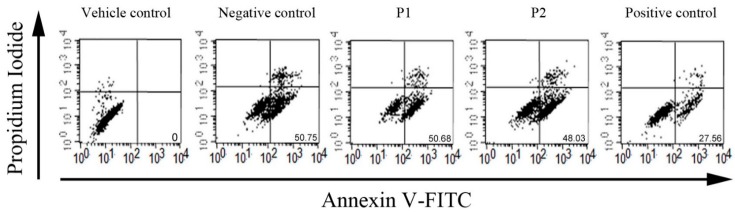
Effect of WBH peptides on H_2_O_2_-induced apoptosis of RCMEC. RCMEC were retreated with 50 µM P1 and P2 for 12 h before exposure to 300 µM H_2_O_2_. Then apoptosis was assayed by annexin V-FITC and PI staining and analyzed by flow cytometry with fluorescence-activated cell sorting.

## Abbreviation

RCMEC: rat cerebral microvascular endothelial cell
ACE: angiotensin-I-converting enzyme
SOD: superoxide dismutase
CAT: catalase
GSH-px: glutathione peroxidase
BHA: butylated hydroxyanisole
BHT: butylated hydroxytoluene
MTT: 3-(4,5-Dimethylthiazol-2-yl)-2,5-diphenyltetrazolium bromide
HHL: hippuryl-histidyl-leucine
HA: hippuric acid

## Appendix

### Detection of Apoptotic Cells by Flow Cytometry

Apoptosis was assayed by annexin V-FITC and PI staining followed by analysis with fluorescence activated cell sorting (FACS, Becton Dickinson, New Jersey, NJ, USA). The method described in the annexin fluorescein isothiocyanate (VFITC)/Propidium iodide (PI) detection kit was followed for this assay. Briefly, the RCMEC were treated as described in Section 3.4.2., and cultured cells (10^6^ cells/mL) were washed with 1× annexin V-FITC binding buffer before staining with 5 μL annexin V-FITC and 10 μL PI for 15 min at room temperature in the dark. Then, 400 μL PBS was added, and the cells were analyzed by flow cytometry.

As shown in Figure A1, the apoptotic rate of RCMEC was increased significantly compare with control group after exposure of the cells to 300 µM H_2_O_2_ for 2 h. Preincubation of the RCMEC with 50 µM of two purified peptides inhibited apoptosis of the cells.
